# Estimate the Unknown Environment with Biosonar Echoes—A Simulation Study

**DOI:** 10.3390/s21124186

**Published:** 2021-06-18

**Authors:** Muhammad Hassan Tanveer, Antony Thomas, Waqar Ahmed, Hongxiao Zhu

**Affiliations:** 1 Department of Robotics and Mechatronics Engineering, Kennesaw State University, Marietta, GA 30060, USA; 2 Department of Informatics, Bioengineering, Robotics, and Systems Engineering, University of Genoa, 16145 Genoa, Italy; antony.thomas@dibris.unige.it; 3 PAVIS, Istituto Italiano di Tecnologia, 16152 Genoa, Italy; waqar.ahmed@iit.it; 4 DITEN, University of Genoa, 16145 Genoa, Italy; 5 Department of Statistics, Virginia Tech, Blacksburg, VA 24061, USA; hongxiao@vt.edu

**Keywords:** Unmanned aerial vehicles, simulation, bio-inspired sensing, biosonar, wavelet scattering, support vector machine

## Abstract

Unmanned aerial vehicles (UAVs) have shown great potential in various applications such as surveillance, search and rescue. To perform safe and efficient navigation, it is vitally important for a UAV to evaluate the environment accurately and promptly. In this work, we present a simulation study for the estimation of foliage distribution as a UAV equipped with biosonar navigates through a forest. Based on a simulated forest environment, foliage echoes are generated by using a bat-inspired bisonar simulator. These biosonar echoes are then used to estimate the spatial distribution of both sparsely and densely distributed tree leaves. While a simple batch processing method is able to estimate sparsely distributed leaf locations well, a wavelet scattering technique coupled with a support vector machine (SVM) classifier is shown to be effective to estimate densely distributed leaves. Our approach is validated by using multiple setups of leaf distributions in the simulated forest environment. Ninety-seven percent accuracy is obtained while estimating thickly distributed foliage.

## 1. Introduction

Unmanned aerial vehicles (UAVs) provide cost-effective alternatives to human labor for a broad variety of tasks such as search, rescue, monitoring and provision. Existing sensing and navigation systems of UAVs, however, have limited capabilities as they either rely on external motion-capture systems, where the perception challenge is eliminated, or only operate in clear, open spaces with sparse obstacles [1,2]. Furthermore, most existing systems are vision-based and therefore only function during daytime or in limited lighting conditions. Active laser-based simultaneous localization and mapping (SLAM) is more accurate and can operate in the dark but usually requires a high payload and produces large amounts of data that are expensive to be processed by small platforms with size, weight and power (SWaP) constraints and limited computational capabilities. It is highly desirable to develop novel sensing and navigation systems that are parsimonious yet suitable for complex, unstructured natural environments under varied lighting conditions.

Echolocating bats demonstrate that it is possible to perform fast, efficient navigation in complex environments with small, low cost transducers and limited onboard computation. Bats employ miniature sonar systems with a few transducers—a nose (or mouth) and two ears—however, they are able to navigate freely through highly unstructured natural habitats such as bushes and forests, often in absolute darkness. They accomplish this by transmitting a chirp signal to the atmosphere and detecting the returned echo signal. With the ability to process two echo signals that are 2 ms apart and to distinguish objects that are 0.3 mm apart [3], they can not only effectively locate objects in 3D but can also recognize the shape and size of objects [4]. This motivates the development of biomimetic sonars, also known as biosonars, for aerial robots. With successful deployment of airborne biosonars, the aerial navigation capability of UAVs can be significantly improved.

In recent years, a great deal of efforts have been devoted to the design of bat-inspired UAV systems to mimic the sensing behavior of bats. For example, Bogue [5] and Ramezani et al. [6] developed biomimetic robots that can perform independent flying by imitating the morphological properties of versatile bat wings. In [7], a autonomous bat-like terrestrial robot was developed that creates a 2D mapping of the environment by using echoes. The authors in [7] developed a robotic prototype and a simulation framework to implement the bat algorithm in swarm robotics. The established bat-inspired navigation systems have been tested on various tasks such as object localization and identification [8,9,10,11], obstacle-avoidance [12,13], tracking [14], landing [15] or convoy control [16].

Despite the progress, existing works have primarily focused on the creation of mechanisms and structures that allow these robots to mechanically attain the agility and mobility of bats. The underlying biosonar sensing paradigm—in particular, how bats achieve efficient mapping and navigation based on biosonar echoes—has been less studied. Additionally, most existing biosonar-based UAV systems focus on achieving certain tasks such as avoiding obstacles and identifying landmarks in the presence of sparsely distributed obstacles—obstacles that are isolated in space. For example, Schillebeeckx et al. [11] focused on localizing reflectors with simple shapes such as balls or blocks; Yamada et al. [12] achieved obstacle avoidance in an environment consisting of randomly distributed plastic poles in a chamber. Studies into bisonar sensing under more complicated, unstructured environments such as trees and forests are limited. Furthermore, due to the constraints in conducting actual experiments, only human-designed laboratory environments or pre-selected natural experimental sites are used for training and testing. For example, in [10,14], landmark tracking is achieved via artificial trees. In [12], a sensing environment is synthesized in a chamber, and in [17], two greenhouses are used as the experimental sites to validate their approach on a terrestrial robot.

In this paper, we present a simulation study for the estimation of foliage distribution as a UAV equipped with biosonar navigates through a forest. In contrast to existing approaches, our study makes several unique contributions: (1) instead of mimicking the mechanisms and structures of bats, we explore the working mechanism of bats’ biosonar through simulation and statistical learning; (2) instead of assuming sparsely distributed obstacles, we focus on unstructured natural environments such as trees and forests that consist of many reflectors; (3) unlike existing research that depends on experimental data, our simulation framework can produce richer sensory data that are not otherwise available under experimental setups, making training and testing inexpensive, flexible, and efficient; (4) instead of focusing on specific tasks such as obstacle avoidance, our simulation framework allows for more sophisticated sensing and navigation scenarios.

In our previous work [18], we built a simulation framework that generates natural sensing environments and produces biosonar echoes under various sensing scenarios. The goal of this paper is to extend our previous work by developing an approach to estimate the environment based on simulated biosonar signals. Specifically, we consider an aerial robot equipped with a biosonar sensor navigating through a forest full of foliage. As the UAV navigates, chirp signals are sent to the trees or to other vegetation, which results in echoes reflected from the environment. We adopt the approach described in [19] and [18] to simulate a random forest consisting of natural looking trees and adopt the bisonar simulator described in [18,20,21] to simulate foliage echos by mimicking a bat’s biosonar. The returned echoes consist of the reflections of chirp signals from various numbers of leaves. Based on these echoes, we aim to estimate the approximate locations as well as the density of leaves. These estimations allows us to synthesize a partial map of the environment, based on which navigation decisions can be made. We distinguish the cases of sparsely and densely distributed tree leaves and adopt different feature extraction and estimation approaches. In particular, a batch processing method is used to estimate sparsely distributed leaf locations; in the case of densely distributed leaves, we employ wavelet scattering to extract features from the echo signals and feed them to a support vector machine (SVM) classifier to estimate the leaf density. Precisely, for efficient navigation, a UAV must understand the environment in real-time. Therefore, our study presents a novel framework for estimating the foliage distribution of the environment in real-time. Once a UAV is equipped with the proposed biosonar-based environment sensing system, any standard navigation protocol can be employed for navigation through the forest.

The rest of the paper is organized as follows. Section 1 entails related work and highlights the significance of our study. In Section 2, we introduce our methods and demonstrate its effectiveness under multiple setups, with a detailed description of our results in Section 3. Section 4 concludes this paper.

## 2. Method and Materials

In this section, we elucidate our estimation approach. We considered a UAV/UAVs equipped with a biosonar navigating in a forest whose map was not known a priori. The UAV was thus required to quickly evaluate the environment for efficient navigation. We set up our sensing environment by first simulating natural looking trees using Lindenmayer systems (L-systems) and 3D Computer-Aided Design (CAD). The simulated trees were then used to form a forest. The random locations of trees were generated from an inhomogeneous Poisson process (IPP) [22]. Details can be found in [18,21]. As the UAV navigated through the simulated forest, its biosonar collected measurements in the form of foliage echoes. These echoes were then used to estimate the location and density of foliage. This process could be performed as needed when the UAV navigates, and partial maps of the forest could be formed by synthesizing estimates from different scans. In this section, we describe two different approaches for foliage estimation—one for sparse leaf scattering and the other for densely distributed leaves.

### 2.1. Batch Processing

In the case of sparsely scattered leaves, we first recorded the impulse responses received by the biosonar as the UAV navigatedw. We considered different hover positions of the UAV around a tree and then recorded the impulse response signals received from the leaves. In each simulation, we also varied the number of leaves randomly. This was performed to generate training data which were then used to estimate certain parameters that helped us to predict the number of leaves that generated the impulses. Below, we discuss this process in detail.

The arrival times of the impulse responses, when multiplied with the speed of sound, provided the travel distance of the emitted sound, which thus could be used to estimate the distance between the sonar and the reflectors. However, the arrival time of the impulse response is not sufficient to determine the leaf locations for two reasons: first, the exact location of a leaf in the sonar beam depends on both the distance to the sonar receiver and the incident angle; there are multiple locations in the sonar beam that have the same distances to the sonar sensor. Second, in most cases there are multiple leaves around a given location; thus, the impulse response is a superposition of reflected waveforms from numerous leaves. As a result, the distance calculated from arrival time alone only gives a rough estimate of the whole group of leaves. We therefore combined arrival times and other features extracted from the impulse response to estimate the leaf locations.

One feature that carries important information about the leaves is the amplitude of impulse responses. For a single leaf, ideally, the number of peaks in the amplitude remains the same irrespective of the leaf location. For a cluster of leaves, the number of peaks will be influenced by the total number of reflectors. However, this information can be leveraged to estimate the number of reflectors in the sonar beam. Specifically, we used a training dataset to learn a mapping between the number of peaks in the amplitude and the number of leaves in the sonar beam. We note here that the beam width and orientation of the sonar beam influences the amplitude of the impulse response. However, we were only interested in the number of peaks in the amplitude, not their actual values. We thus normalized the amplitudes by dividing them by the maximum value. The nonzero amplitudes were first extracted by imposing a threshold on the impulse response signal, based on which the number of peaks was counted.

We thus employed two features to estimate the leaf distribution: the arrival times $t_{i}$ and the number of amplitude peaks $p_{i}$. Each $t_{i}$ is the arrival time of the maximum of peaks, which was used to estimate the distance between the sonar and the leaf cluster. We denote the estimated distance by $d_{i}^{max}$. To estimate the actual number of leaves $n_{i}$, we assumed a general mapping of the form $n_{i}=f\left(p_{i}\right)+\epsilon_{i}$. Specifically, we assumed a linear model $n_{i}=\alpha p_{i}+\beta +\epsilon_{i}$, i=1,⋯,n, with *n* being the number of training samples. Note that the linear assumption was chosen based on extensive training and testing compared with alternative linear or nonlinear models including the generalized linear model, Gaussian process regression model and generalized additive model. Based on this model, we performed least-squares estimation, also called batch processing [23], to obtain the best estimate of α and β. Once the two estimates were computed, they could be used to predict the number of leaves given $p_{i}$. Let ${\hat{n}}_{i}$ denote the predicted leaf number; we further estimated the leaf locations by sampling ${\hat{n}}_{i}$ leaf locations from a Gaussian distribution, denoted by $N(c_{i},\sigma_{i})$. We set the center of the Gaussian distribution $c_{i}$ to be the location on beam center with distance $d_{i}^{max}$. To obtain the standard deviation for the Gaussian distribution $\sigma_{i}$, we also computed leaf distances based on the arrival times of the minimum peak values. Since there were two possible distances, we selected the location closest to the sonar location and denoted the distance as $d_{i}^{min}$. Note that $d_{i}^{min}$ could be computed as we knew the number of peaks as well as the arrival times of peaks. The standard deviation was then computed by $\sigma_{i}=\left|\right|d_{i}^{max}-d_{i}^{min}\left|\right|/2$, where ||·|| denotes the standard Euclidean norm. The sampled leaf locations were then utilized to construct the map.

From our experience, for all practical purposes, a random sampling from the Gaussian distribution produces a good approximation of the actual leaf locations. We also would like to note that, when performing the training and prediction, we divided the sonar beam into a few segments and treated the impulse response signals within each segment separately. This not only produced richer training data but also allowed us to obtain a more accurate prediction of different regions of the sonar beam.

### 2.2. Data Structure and Prepossessing

The received echo signal consisted of varied magnitudes and lengths of impulses depending upon the number of leaves and their exposed surface area intercepted by the transmitted lobe. A few example raw echo samples representing distributed leaf reflections are shown in result section, with the number of leaves varying from 1 to 12. Moreover, the time interval between impulse envelopes in a received echo signal also varied. Though such discontinuity reflected the structure of the leaf cluster, it had a trivial correlation with the leaf density intercepted by the lobe.

We addressed these challenges by (i) removing dead zones—signal values in between $-10^{-3}$ and $10^{-3}$ that resulted in a significant data dimensionality reduction; i.e., up to a maximum sample length of 4096 instead of 8192 raw data points—-and (ii) re-scaling the varying length of truncated data samples by means of up/down sampling to a fixed length of 4096 data points.

### 2.3. Representation Learning

**Figure 1:**
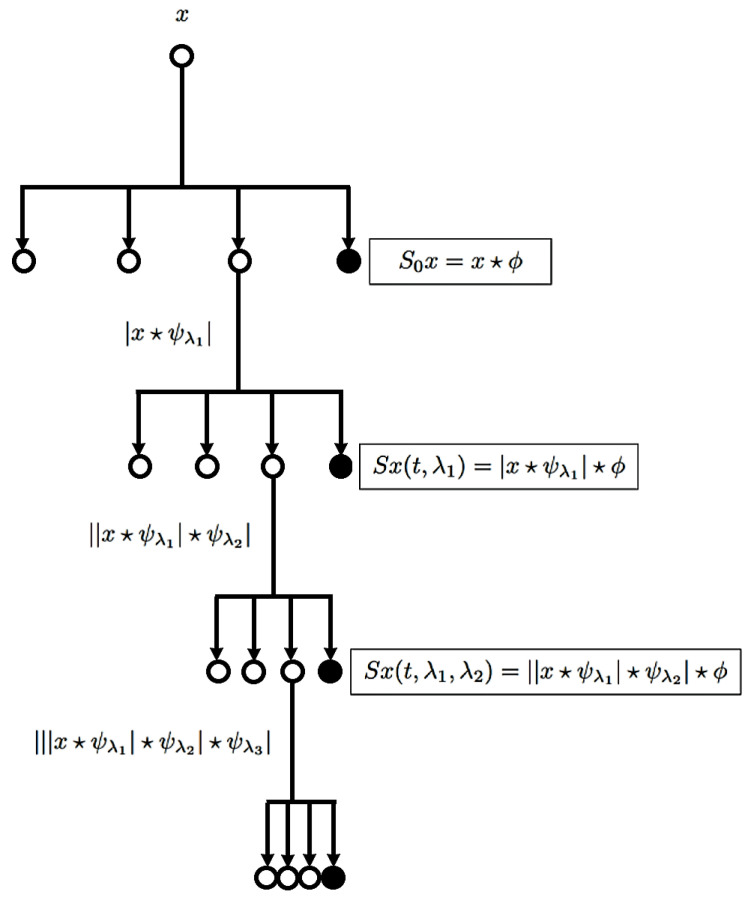
A wavelet scattering transform iterates on a set of wavelet modulus operators to compute cascades of wavelet convolutions and moduli, generating averaged scattering coefficients.

In this study, we employed the wavelet scattering transform (WST) [24] to extract discriminant features from the pre-processed echo signals. Specifically, WST refers to an iterative process of applying a set of wavelet transforms and nonlinearities at different scales, making it stable in the case of small deformations and invariant to input signal translations/rotations. As shown in Figure 1, the operations necessary for the transformation of the input signal **x** into a wavelet are (1) convolution, (2) nonlinearity and (3) averaging. Precisely, the WST coefficients are obtained by applying the convolution operator ⋆ between the wavelet modulus and low-pass filter ϕ. Assuming wavelet ψ(t) to be a band pass filter with a central frequency normalized to 1 at time index *t*, the wavelet filter bank $\psi_{\lambda }\left(t\right)$ is defined as follows:

(1) $$\begin{array}{c} \psi_{\lambda }\left(t\right)=\lambda \psi \left(\lambda t\right), \end{array}$$

where $\lambda =2^{\frac{j}{N_{w}}}$, ∀j∈Z considering $N_{w}$ as the number of wavelets per octave. The term $1/N_{w}$ represents the order of the wavelet bandwidth ψ(t); thus, the filter bank is composed of band pass filters with a frequency bandwidth of $\lambda /N_{w}$ (centered in the frequency domain defined by λ).

At the initial stage, we had a single coefficient, defined as

(2) $$\begin{array}{c} S_{0}x\left(t\right)=x⋆\phi \left(t\right), \end{array}$$

which for our echo signals was close to zero. By averaging the wavelet module with ϕ, the following coefficients were obtained:

(3) $$\begin{array}{c} Sx\left(t,\lambda_{1}\right)=\left|x⋆\psi_{\lambda_{1}}\right|⋆\phi \left(t\right) \end{array}$$

The coefficients of the second order were used to detect the high-frequency amplitude modulation of each frequency band in the first layer.

(4) $$\begin{array}{c} Sx\left(t,\lambda_{1},\lambda_{2}\right)=||x⋆\psi_{\lambda_{1}}\left|⋆\psi_{\lambda_{2}}\right|⋆\phi \left(t\right). \end{array}$$

Since the wavelets $\psi_{\lambda_{2}}$ with an octave resolution of $N_{W_{2}}$ can be different from $N_{W_{1}}$, we set $N_{W_{2}}=1$ for echo signals with more time support to define wavelets. Consequently, we obtained a sparse, deep representation concentrating on a small number of wavelet coefficients for which the low-pass ϕ filter ensured local invariance to echo signal time shifts.

Figure 1 shows the topology of the wavelet scattering coefficients resembling the structure of deep neural networks (except for the fact that WST provides intermediate outputs). Features from the second order were normalized to ensure that the higher order of scattering depended on the amplitude of the echo signal component. A scattering feature vector for a particular frame was concatenated in both the first and second orders of WST. Furthermore, to ensure frequency translation invariability, the scattering preserved more detail in the echo signal by employing log-mel to each higher-order feature vector coefficient. Thus, stable deformations along with the local translation invariability were achieved in both the time and log frequency domains.

Specifically, to obtain wavelet scattering coefficients of pre-processed echo signals, we used an invariant scale of 0.22 s and an over-sampling factor of 2. Consequently, the resulting feature dimension of each data sample was n=211, which was then used to train and evaluate the classification model.

### 2.4. Classifier

In order to estimate the density of leaf clusters, we employed the Support Vector Machine (SVM), which is one of the most powerful supervised machine learning algorithms. The key advantage of using the SVM is that, unlike many other machine learning algorithms such as neural networks, the SVM does not require extensive hyper-parameter tuning to obtain good results. The SVM offers low inference latency and thus is suitable for classification in real-time. This performant algorithm determines the optimal hyperplane by handling non-linearly separable data using kernels. Precisely, neural networks find the hyperplane by iteratively updating its weights and trying to minimize the cost function. It produces an optimal hyperplane that could best separate the data—a hyperplane in which the margin between the decision boundary and the closest data points is maximized.

For a given dataset, $D={\left\{\left(x_{i},y_{i}\right)\;|\;x_{i}\in R^{n},y_{i}\in \left\{-1,1\right\}\right\}}_{i=1}^{m}$, where *n* represents the dimensionality of the input data, and *m* represents the total number of samples, consider the equation of the hyperplane w·x=−b, where w represents the trainable parameters of the SVM classifier. Thus, if the point (**x**,y) is on the hyperplane, w·x+b=0; if the point (**x**,y) is not on the hyperplane, the value of w·x+b could be either positive or negative (the two classes considered for binary classification problem optimization). To achieve better performance for our challenging non-linearly separable echo signal data, we employed the well known Radial Basis Function (RBF) kernel defined by $K\left(x_{i},x_{j}\right)=exp(-\gamma ||x_{i}-x_{j}{\left|\right|}^{2})$. The RBF kernel is also called the Gaussian kernel. It can learn complex decision boundaries. The RBF kernel contains a parameter γ. A small value of γ will cause the model to behave like a linear SVM. A large value of γ will mean that the model is heavily impacted by the support vector examples.

However, the SVM was primarily designed for binary classification. For its extension to a multi-class classification framework, in this study, a one-versus-the-rest approach (also known as one-vs-all) was used to estimate the number of leaves considering various classes. Thus, we iterated over c∈C classes and treated the data from a particular class *c* as its positive class and all the other classes as one negative class. Consequently, such a binary classification formulation reduced the distance between data point *x* and the actual class *c* while maximizing the distance of *x* from all the other classes.

## 3. Results and Discussion

**Figure 2:**
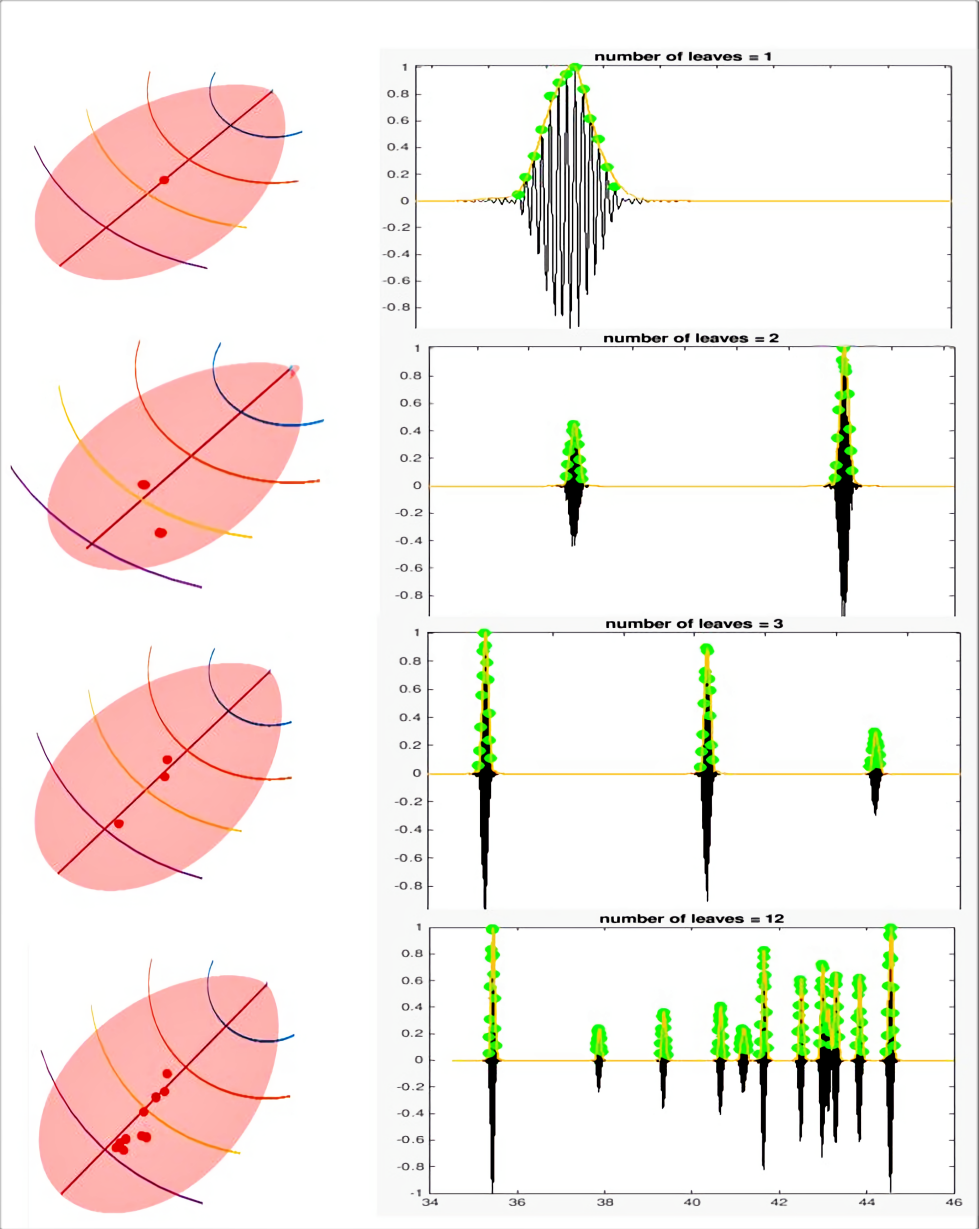
The main lobe was divided into five equally spaced segments using hyperplanes. The leaf position can be seen on the left side. The right side of the plots shows the recorded echo peaks and their time spans. Here, the *X*-axis denotes the time span and the *Y*-axis denotes the amplitude of the recorded signal.

In this section, we show the validation of our proposed approach by means of a simulation framework. All the experiments were carried out with an Intel^®^ 2 × 16 Core™ E5-2683v4 Broadwell processor 2.1 GHz and 128 GB Intel processors, 2400 MHz RAM with Ubuntu 16.04 LTS in the MATLAB environment. We performed a pilot study by designing a simple sensing scene that involved multiple trees and a UAV (as a bat) navigating across them. The trees were constructed by combing an extended L-system with CAD-developed object files as described in [18]. We took two scenarios into account: (1) the leaves were sparsely distributed, and (2) the leaves were densely distributed. In each simulation, the impulse responses of a virtual biosonar sensor were collected and analyzed. In various sites of the field, impulse responses were calculated to imitate a hovering quad-rotor. In Figure 2, we illustrate several examples of the sparsely distributed leaves. The main lobe was selected to have a bandwidth of 30 dB.

A few simulation setups for the sparsely distributed leaves are demonstrated in Figure 2. On the left column of Figure 2, we show four different leaf distribution setups, with the number of leaves in the main lobe varying from 1 to 12. In each setup, we repeated the simulation 10 times by randomly selecting the location of the leaves in the main lobe. The main lobes in each of the four cases were divided into five segments separately by hyperplanes that contained points with equal distances to the sonar receiver. As elucidated in Section 2, we estimated $\alpha_{i}$, $\beta_{i}$ using the training data from each of these segments. The estimated values were then averaged to obtain the required parameters α and β, which were then used to estimate the locations of leaves. On the right column of Figure 2, we mark the peaks of the amplitude on the impulse response signals. Thus, to generate ground-truth labels, the leaf density (number of leaves in the lobe) was estimated using our proposed batch processing approach, as elucidated in Section 2.

**Figure 3:**
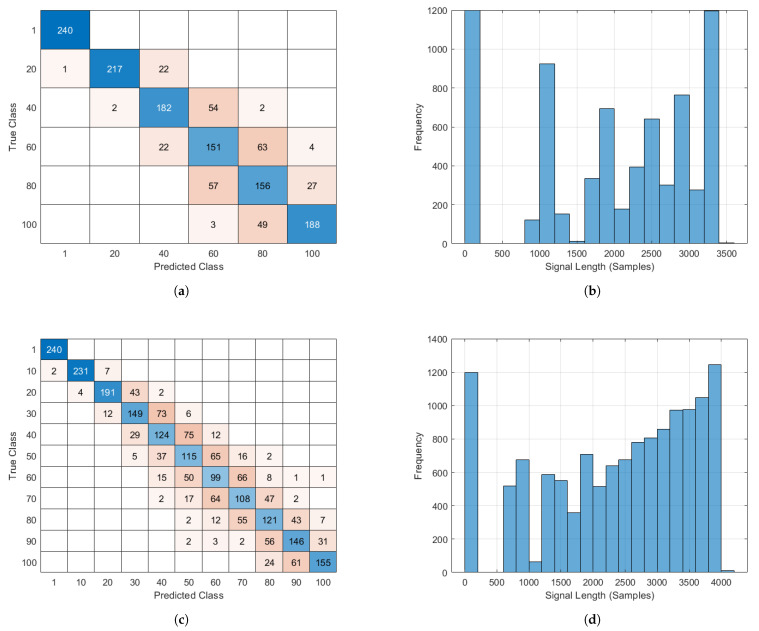
Wavelet scattering for feature extraction using the SVM as a classifier. (**a**) Confusion chart with train set, 5040 samples; validation set, 240 × 6 = 1440 samples; test set, 1080 samples; test accuracy 78.75%; (**b**) trimmed echo signal histogram that represents the set of data used in experiments. There were a total of 7200 samples of six classes with lengths measured in milliseconds; (**c**) Confusion chart with train set, 9240 samples; validation set, 240 × 11 = 2640 samples; test set = 1980 samples; test accuracy 74.15%; (**d**) Trimmed echo signal histogram that represents the set of data used in experiments. There were a total of 13,200 samples of 11 classes with lengths measured in milliseconds.

The overall dataset comprised 13,200 data samples representing 11 classes (1200 samples per class). We varied the number of leaves that fell inside the main lobe of the sonar from 0 to 100 and evaluated two scenarios of different discrete resolutions: 10 and 20 leaves, respectively. For example, in Figure 3a, the leaves were varied from 0 to 100 with an increment of 20 leaves. In this particular setting, we aimed to classify input samples as having either 1, 20, 40, 60, 80 or 100 leaves in the lobe. Consequently, we had 7200 samples in total, out of which we randomly picked 5040, 1440 and 1080 samples and treated them as training, validation and testing sets, respectively. In Figure 3b, a histogram of the signal length of the data samples is presented, where the dead points ($-10^{-4}$ < signal-strength < $10^{-4}$) from the raw echo signal were removed to extract a trimmed/finer version of the data. Consequently, echo samples of varied lengths, each representing a particular class, constituted the proposed dataset. Similarly, for the resolution of 10 leaves (see Figure 3c,d), we aimed to classify input samples as having either 1, 10, 20, 30, 40, 50, 60, 70, 80, 90 or 100 leaves in the lobe. Consequently, we had 13,200 sample in total, out of which randomly picked 9240, 1980 and 1980 samples as training, validation and testing sets, respectively.

**Figure 4:**
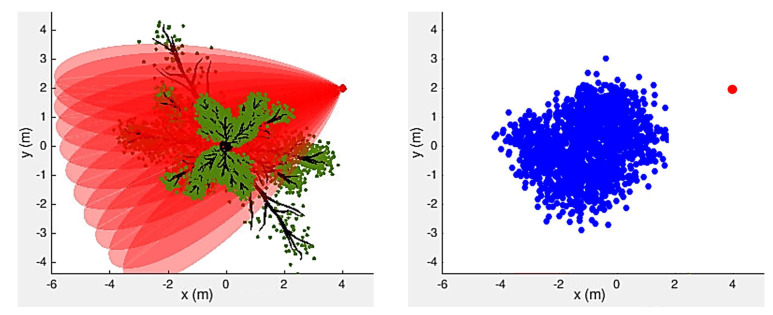
In dense mode—the recorded echo peaks and time span show the number of leaves in the diverse environment—a map is created with x,y positions in meters.

However, with a growing leaf density, merely counting the number of spikes in the echo signal could not precisely reflect the leaf density in the sparse mode. In Figure 4, we demonstrate an example of this. The left panel of Figure 4 shows a bird’s-eye view of the scene, consisting of a single tree and a biosonar, and the right panel shows the reconstructed map based on our target estimation result. As illustrated in the left panel of Figure 4, to collect data, the UAV scanned its surroundings from top to bottom and from left to right with intervals of 5 degrees. The resulting impulse responses were used to predict the number of leaves. Therefore, we made use of Machine Learning techniques to predict the number of leaves in the lobe.

Specifically, based on the wavelet scattering coefficients, we employed the SVM with a RBF as the kernel function to train a classification model on the acquired dataset. We used an invariant scale of 0.22 s when performing wavelet time scattering. Since the scattering transformation was averaged for all time samples, w set the over sampling factor to 2, meaning that the number of scattering coefficients on each path increased by four in relation to the critically down-sampled values. The pre-processing stage truncated or padded the raw data sample up to a maximum length of 4096 data points. We kept the model parameters fixed for all the experiments pertaining to each setting; i.e., 6 and 11 classes with a leaf density resolution of 10 and 20, respectively.

To evaluate our proposed approach, we considered a UAV equipped with a biosonar navigating in a simulated forest environment. The reported results show that the proposed method achieved promising performance in both settings; i.e., 78.75% and 74.15% accuracy for 6 and 11 classes, respectively. Precisely, the cases in which our model predicted a smaller number of leaves than the actual count (ground-truth) corresponded to the clusters where all the leaves were not fully exposed, instead exhibiting significant overlap. The training data samples with overlaps also affected the model, causing it to predict a higher number of leaves in some cases. Nevertheless, the misclassification cases followed a diagonal pattern that resulted in a fair estimate of the leaf density. From another perspective, such an estimation capability imitates the natural apprehension of bats, who roughly estimate the density of leaf patches instead of counting every leaf to gain an understanding in order to make decisions.

## 4. Conclusions

In this paper, we have discussed an approach for synthesizing an environment from a bat biosonar sensor by predicting the number of leaves on trees. Two different leaf density patterns, both sparse and thick, were considered using our approach. To this end, we employed two different estimation techniques for leaf prediction: one for the sparse distribution and another for the dense distribution. Since leaf density estimation is a challenging task due to the irregular exposure of leaves, we employed Machine Learning techniques to generalize in varying scenarios, which functioned well. The validation of our approach under a simulation framework showed promising results for application in real-world scenarios.

We adopted the biosonar simulator developed by [18,20,21] to simulate foliage echos. It is worthwhile to mention that natural environments can have many reflectors other than leaves, such as tree trunks, branches, rocks, etc. The relative importance of these different scatterers will depend on the environment. As discussed by [18,25], in a forest environment, very little of the echo comes from branches, and most of the returned echo originates from leaves. Therefore, we believe it is a practically valid assumption that leaves remain the major source of reflectors under this setup.

In this work, we considered a hovering UAV while receiving the impulse responses. We intend to extend our approach to the general case of navigating UAVs. As future work, we also expect to extend our approach to a multi-robot setting. With multi-robot communication, we envisage a better efficacy of leaf estimation.

The software developed in this paper will be made available as an open source package on the Github site (https://cfpss.github.io/, accessed on 30 June 2021) upon acceptance of this manuscript. 

## Data Availability

Not applicable.
